# Impact of an Intuitive Eating Intervention on Disordered Eating Risk Factors in Female-Identifying Undergraduates: A Randomized Waitlist-Controlled Trial

**DOI:** 10.3390/ijerph191912049

**Published:** 2022-09-23

**Authors:** Julia A. Katcher, Richard R. Suminski, Carly R. Pacanowski

**Affiliations:** Department of Behavioral Health and Nutrition, University of Delaware, Newark, DE 19716, USA

**Keywords:** body image, disordered eating, dietary restraint, intuitive eating, college students, feeding and eating disorders

## Abstract

Dietary restraint and low body appreciation are common among female-identifying undergraduates and are related to the development of disordered eating, which female-identifying undergraduates engage in throughout college. Training students in intuitive eating, an approach that promotes eating by internal cues, may be a way to ameliorate dietary restraint and low body appreciation, ultimately decreasing disordered eating. The purpose of this study was to examine the impact of a five-week intuitive eating intervention on dietary restraint, body appreciation, and intuitive eating in female-identifying undergraduates. A five-week intuitive eating intervention (NCT0394700) was facilitated by two Registered Dietitians. One treatment group (TG) (*n* = 7) and one waitlist control group (WLCG) (*n* = 7) participated in the trial. From baseline to post-intervention, there was a significant decrease in dietary restraint, *t*(12) = −2.88, *p* = 0.01, and a significant increase in intuitive eating, *t*(12) = 4.03, *p* = 0.002, in the TG compared to the WLCG. The intervention had replicable effects on all outcome variables. Measurements at the five-week follow-up suggested the impact was sustained. This study provides preliminary data suggesting an intuitive eating intervention may help improve disordered eating risk factors by decreasing dietary restraint and increasing intuitive eating in female-identifying undergraduates.

## 1. Introduction

Many college students utilize dieting as a means of weight loss, and studies have found that female-identifying undergraduates engage in disordered eating behaviors, such as unhealthy weight control methods, throughout college. A study of 638 college students found that 22% of students were eating according to a weight loss diet, 3% were fasting or consuming diet pills, 2% were chewing and spitting out their food, purging, or taking laxatives, and 1% were using diuretics [1]. Another study found that a significant proportion of the college population engaged in excessive exercise and binge eating. The behavior that most strongly predicted disordered eating was one’s desire to lose weight [2]. A large study assessing disordered eating among students at 12 U.S. colleges and universities found that 11% of the sample had a high risk for developing an eating disorder, 40% reported binge eating, and 30% reported compensatory behaviors through purging, laxatives, diuretics, diet pills, or overexercising [3]. The proportion of students eating according to a weight loss diet raises concerns since dieting in adolescents can predict outcomes related to eating disorders up to five years later [4]. Disordered eating, which differs from an eating disorder meeting specific diagnostic criterion, encompasses various eating issues, such as, restraining dietary intake and binge eating [5,6]. Female-identifying undergraduates commonly report disordered eating [6]. Data show that first-year female-identifying undergraduates have reported increased disordered eating over their freshman year [7]. Taken together, these findings suggest that many of the eating and weight control behaviors used by college students are problematic. Two risk factors that are important targets in preventing disordered eating are high dietary restraint and low body appreciation.

Dietary restraint refers to the cognitions (e.g., cognitive restraint) related to the intention of restraining caloric intake to manipulate one’s body weight [8,9,10]. These cognitions, when combined with other risk factors, can increase one’s risk for the development of eating disorders and adverse psychological effects, such as obsessive worry about weight and body shape, in high-risk individuals [10,11,12,13,14]. One study in female-identifying undergraduates concluded that dietary restraint prospectively predicted increases in disordered eating over the first year of college [7]. Therefore, dietary restraint is a potentially important target for disordered eating prevention efforts in female-identifying undergraduates.

Body appreciation has been identified as a protective factor in the development of eating disorders [15] and is a component of positive body image that focuses on respecting, accepting, approving, and thinking favorably about one’s body [16]. High body appreciation scores have been strongly, negatively associated with and related to body dissatisfaction and disordered eating, respectively [16], and body dissatisfaction and dietary restraint are significant longitudinal predictors of disordered eating outcomes [17]. Since body appreciation may be protective in the development of disordered eating and body appreciation has a negative relationship with eating disorder symptomatology, further investigation is warranted. This is particularly relevant in female-identifying undergraduates with high dietary restraint and low body appreciation and are likely to attempt to lose weight through methods such as dieting.

An alternative approach to dieting that encourages a healthy relationship with food and may improve disordered eating risk factors is intuitive eating. Intuitive eating is a non-restrictive way of eating based on ten principles, which promote listening to one’s internal cues (e.g., hunger and fullness) and body acceptance. Intuitive eating focuses on eating enjoyable and nutritious foods and respecting one’s health [18]. Several reviews and meta-analyses have been published assessing the psychosocial correlates of intuitive eating and have found that intuitive eating was significantly negatively associated with dietary restraint and significantly positively associated with body appreciation [19,20]. Intuitive eating and body appreciation were explored together as advised by the co-author of intuitive eating, Evelyn Tribole [18], and research supporting the connection between intuitive eating and body appreciation. The acceptance model of intuitive eating is based on the associations between body appreciation, intuitive eating, body acceptance by oneself and others, and resistance to adopting an observer’s perspective of one’s body. This model posits that body appreciation predicts intuitive eating and that favorable body attitudes are associated with higher awareness of internal signals, such as hunger and fullness cues, and a greater tendency to honor these cues [21]. A population-based study in young adults found that individuals who reported that they trust their body to tell them how much food to eat had lower odds of engaging in disordered eating behaviors such as binge eating, laxative use, skipping meals, and dieting [22]. Despite intuitive eating’s promising results on disordered eating behaviors, few studies have conducted randomized controlled trials that adhere to the intuitive eating principles outlined by Tribole and Resch and assess intuitive eating interventions in the college population. Burnette and Mazzeo conducted the first intuitive eating intervention that followed the principles outlined by the developers, Tribole and Resch, in college women. This study found reductions in disordered eating behaviors and body dissatisfaction and improvements in body appreciation and intuitive eating [23]. However, this trial was not controlled and did not utilize Registered Dietitians and Certified Intuitive Eating Counselors as interventionists. Another non-dieting intervention was conducted in college women and found improvements in intuitive eating, dieting intention, and body image dissatisfaction [24]. However, it is unclear if intuitive eating was the primary focus of the intervention content and if the intervention followed the principles outlined by the developers [18]. Additionally, it is important to focus intuitive eating interventions on individuals who identify as female because they typically have higher levels of restrained eating compared to individuals who identify as male [14], in addition to the disordered eating behaviors mentioned above, which are more prevalent in female-identifying undergraduates.

Given the lack of randomized controlled intuitive eating intervention studies that adhere to the intuitive eating principles outlined by Tribole and Resch conducted in female-identifying college students, more research is warranted to extrapolate intuitive eating’s effects. The present study aimed to examine the impact of a five-week randomized waitlist-controlled intuitive eating intervention on disordered eating risk factors, dietary restraint and body appreciation, in female-identifying undergraduates. It was hypothesized that an intuitive eating intervention would decrease levels of dietary restraint and increase body appreciation and intuitive eating and that these effects would be replicable in a waitlist control.

## 2. Materials and Methods

### 2.1. Study Organization and Eligibility Criteria

This study utilized a five-week curriculum for teaching intuitive eating in a group setting created by a Registered Dietitian [25]. This curriculum was based on the original book, *Intuitive Eating: A Revolutionary Program that Works*, written by two Registered Dietitians [18]. Permission was granted to modify and test the curriculum within a college population in which, to our knowledge, it had not yet been tested. Minor modifications were made to the curriculum to ensure the language and examples were appropriate for the college-aged population. Two Registered Dietitians trained under intuitive eating expert Evelyn Tribole to become Certified Intuitive Eating Counselors. Before conducting the sessions, the Registered Dietitians/Certified Intuitive Eating Counselors reviewed the curriculum for content validity. Each session reviewed 1–3 intuitive eating principles, incorporated an activity to aid learning the principles, included group discussions, and concluded with 5–10 min of journaling and meditation. Table 1 outlines the weeks, principles taught, and activities used in each session.

Participants were recruited through flyers placed around campus, distributed through email announcements to classes, clubs, and sororities, and shared in courses with high undergraduate enrollment. Eligibility criteria included individuals who were: female-identifying undergraduate students, between the ages of 18–26 years old, scored high on measures of dietary restraint (4.0 or higher), scored low on measures of body appreciation (lower than 3.0), and were available during the scheduled intervention time for the entire five-week intervention. This study excluded anyone who self-reported having an eating disorder in addition to not meeting eligibility criteria.

### 2.2. Study Procedures and Research Tool

The Institutional Review Board approved all study elements before conducting the research and this trial was registered on clinicaltrials.gov (NCT0394700). This intervention utilized a randomized waitlist-controlled study design. Interested individuals (*n* = 60) completed a screening questionnaire. Eligible participants (*n* = 24) were randomly assigned to one of two groups before the baseline assessment using simple randomization via a virtual coin flipper and scheduled to attend the baseline assessment. Those who attended the baseline assessment (*n* = 15) provided written consent to participate in the trial before beginning the assessment. Participants were informed that this study was assessing eating behavior and body appreciation in female-identifying undergraduates. The treatment group (TG) began a five-week intervention immediately after the baseline assessment, and the waitlist control group (WLCG) started an identical intervention five weeks later, after the TG had concluded. Participants were compensated up to $45 for attending all five intuitive eating sessions.

**Figure 1:**
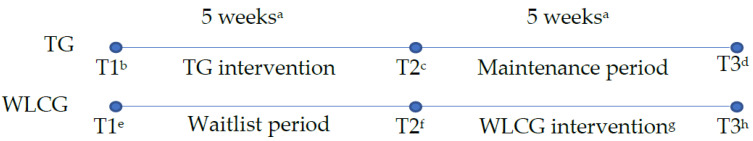
Intervention timeline and assessment time points for the treatment group (TG) and waitlist control group (WLCG).^a^ 5 weeks indicates the length of the intervention period. ^b^ T1 for the TG represents the baseline assessment. ^c^ T2 for the TG represents the post-intervention assessment. ^d^ T3 for the TG represents the maintenance period. ^e^ T1 for WLCG represents the waitlist period. ^f^ T2 for WLCG represents the baseline assessment. ^g^ Same intervention as the TG. ^h^ T3 for WLCG represents the post-intervention assessment.

A questionnaire was administered at three time points, denoted T1, T2, and T3. The TG had a baseline at T1, post-intervention at T2, and maintenance period at T3. WLCG had a waitlist period at T1, baseline at T2, and post-intervention at T3. See Figure 1 for the study design and timeline for each group. Participant age, height, and weight were collected during the baseline assessment. Dietary restraint, body appreciation, and intuitive eating were measured at each time point.

The Three-Factor Eating Questionnaire-r18 is an 18-item self-report questionnaire, of which six questions assess an individual’s level of dietary restraint through a cognitive restraint subscale [26]. This questionnaire contains three subscales: Cognitive Restraint, Uncontrolled Eating, and Emotional Eating, while this study only utilized the Cognitive Restraint subscale. For the Cognitive Restraint subscale, items 2, 11, and 12 are rated on a 4-point scale ranging from 4 (“Definitely True”) to 1 (“Definitely False”). Item 15 is rated on a 4-point scale ranging from 1 (“Almost Never”) to 4 (“Almost Always”). Item 16 is rated on a 4-point scale ranging from 1 (“Unlikely”) to 4 (“Very Likely”). Item 18 is rated on an 8-point scale from 1 (no restraint in eating) to 8 (total restraint in eating). Higher scores indicate greater cognitive restraint [27]. Cronbach’s alpha for the Cognitive Restraint subscale in the current sample at baseline was 0.78. The Three-Factor Eating Questionnaire-r18 has shown adequate validity and reliability in undergraduate women [28].

The Body Appreciation Scale-2 is a 10-item self-report questionnaire developed to assess an individual’s level of positive body image. Items are rated on a 5-point scale ranging from 1 (“Never”) to 5 (“Always”). A mean score is calculated, with higher scores indicating greater positive body image [29]. Cronbach’s alpha for the Body Appreciation Scale-2 in the current sample at baseline was 0.90. The Body Appreciation Scale-2 has shown internal consistency, test–retest reliability, and convergent, incremental, and discriminant validity without intervention in college women [29].

The Intuitive Eating Scale-2 is a 23-item self-report questionnaire developed to assess intuitive eating. This questionnaire contains four subscales: Unconditional Permission to Eat, Eating for Physical Rather than Emotional Reasons, Reliance on Hunger and Satiety Cues, and Body-Food Choice Congruence, while this study utilized the total intuitive eating score. Items are rated on a 5-point scale ranging from 1 (“Strongly Disagree”) to 5 (“Strongly Agree”). Certain items are reversed scored. Higher scores on this measure indicate greater levels of intuitive eating [30]. Cronbach’s alpha for the total Intuitive Eating Scale-2 score in the current sample at baseline was 0.85. The Intuitive Eating Scale-2 has shown internal consistency, test–retest reliability, and construct, discriminant, and incremental validity without intervention in college women [30].

Participants’ height and weight were measured by trained research assistants using standard anthropometric techniques. A portable stadiometer (SECA Model 217, SECA Corp., Hamburg, Germany, 2014) and a research-grade body weight scale (Tanita electronic scale BWB-800S) were used. Weight and height were measured using the Centers for Disease Control and Prevention National Health and Nutrition Examination Survey Anthropometry Procedures Manual [31]. Body Mass Index (kg/m^2^) was calculated from height and weight measures and used to assess baseline differences in the groups, as the intuitive eating curriculum does not discuss weight [18].

### 2.3. Statistical Compilation

Independent samples *t*-tests were conducted to examine differences in baseline characteristics between the TG and WLCG. Three questions were answered to evaluate intervention effects. Question 1 assessed between-groups differences and questions 2 and 3 assessed within-subjects changes.

Question 1: “Are the changes in outcome variables during the intervention in the TG significantly different from the changes in the outcome variables during the waitlist control period in the WLCG?” Analysis of covariance (ANCOVAs) tested for significant between-groups effects (TG vs. WLCG) at T2 on dietary restraint, body appreciation, and intuitive eating, controlling for differences in baseline characteristics. It was hypothesized that there would be significant differences between the TG and WLCG at T2, with the TG seeing significantly greater improvements for all outcome variables.

Question 2: “Are the intervention effects maintained at the five-week follow-up in the TG?” One-way repeated measures analysis of variance (ANOVAs) were independently run to compare within-subjects changes of outcome variables (dietary restraint, body appreciation, intuitive eating) at T1, T2, and T3 in the TG. It was hypothesized that the changes in outcome variables would significantly improve for the TG from T1 to T2 and from T1 to T3 but not from T2 to T3, meaning, the significant effects of the intervention would be maintained from post-intervention to the 5-week follow-up.

Question 3: “Did the intervention produce similar changes in outcome variables during the intervention in the WLCG?” That is, were the intervention effects replicated? A paired samples *t*-test examined within-subjects differences of outcome variables from T2 to T3 for the WLCG. It was hypothesized that changes in outcomes in response to the intervention would be significant for the WLCG, meaning, the intervention would have hypothesized replicable effects in a WLCG.

Given the importance of the results relying on the outcomes of the individual tests themselves and the exact *p*-value for each individual test was reported and discussed appropriately, Bonferroni corrections were deemed unnecessary [32]. Effect sizes were calculated using Cohen’s *d* guidelines: 0.2 indicates small effect, 0.5 medium effect, and 0.8 large effect [33]. The data were evaluated for normality using skewness statistics and the significance level was set a priori at *p <* 0.05. Analyses were performed with IBM Statistical Package for the Social Sciences Statistics Standard version 28.0 for Mac (SPSS version 28.0, SPSS Inc., Chicago, IL, USA, 2021).

## 3. Results

**Figure 2:**
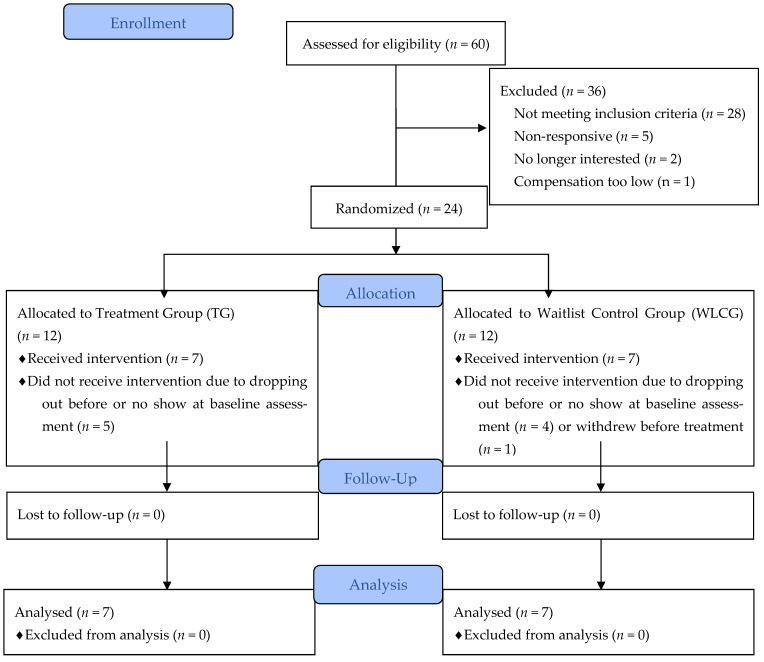
CONSORT diagram of female-identifying undergraduate participants and eligibility status for five-week intuitive eating intervention.

One participant withdrew from the study after attending the baseline assessment and consenting due to a schedule conflict. One TG (*n* = 7) and one WLCG (*n* = 7) received the intervention. The WLCG received the intervention after their T1 and T2 scores were analyzed as an untreated comparison for the TG. See Figure 2 for the CONSORT flow diagram. Participant ages ranged from 19 to 26 years (*M* = 20.9, *SD* = 1.9) and body mass index ranged between 19.9 and 41.6 kg/m^2^ (*M* = 26.4, *SD* = 6.0). At the initial assessment, despite randomization, there was a significant difference in body mass index between the TG (*M* = 22.4, *SD* = 1.5) and WLCG (*M* = 30.3, *SD* = 6.2) [*t*(12) = −3.30, *p =* 0.01]. The magnitude of the differences in means was large [*M* = −7.9, 95% CI (−13.6, −2.19), Cohen’s *d* = −1.76]. The TG contained participants with a body mass index classified as “normal”, and the WLCG contained higher-weight individuals [34]. BMI was added as a covariate to the ANCOVAs. There were no statistically significant differences between groups on other variables. Table 2 details the means and standard deviations of the difference scores for outcome variables for the TG and WLCG from T1 to T2 and T2 to T3.

### 3.1. Question 1: Are the Changes in Outcome Variables during the Intervention in the TG Significantly Different from the Changes in the Outcome Variables during the Waitlist Control Period in the WLCG?

After adjusting for baseline BMI, there were no significant differences between the TG and WLCG on post-intervention dietary restraint, F (1, 11) = 0.007, *p* = 0.934, partial eta squared = 0.001, body appreciation, F (1, 11) = 0.35, *p* = 0.566, partial eta squared = 0.03, or intuitive eating, F (1, 11) = 0.3989, *p* = 0.071, partial eta squared = 0.266.

### 3.2. Question 2: Are the Intervention Effects Maintained at the Five-Week Follow-Up in the TG?

#### 3.2.1. Dietary Restraint

There was a significant effect for time in an ANOVA with dietary restraint score as the dependent variable, Wilks’ Lambda = 0.28, F (2,5) = 6.55; *p =* 0.04, multivariate partial eta squared = 0.72. T1 dietary restraint score (*M* = 64.3, *SD* = 21.0) was higher than T2 (*M* = 42.1, *SD* = 20.8; *p =* 0.06) and T3 (*M* = 46.0, *SD* = 20.0; *p =* 0.02). The T2 score was not significantly different than the T3 score (*p =* 1.00). This suggests that the intervention promoted a significant decrease in dietary restraint scores within the TG and that the decrease was maintained at the five-week follow-up.

#### 3.2.2. Body Appreciation

There was no significant effect for time in an ANOVA with body appreciation score as the dependent variable, Wilks’ Lambda = 0.45, F (2, 5) = 3.06; *p =* 0.14, multivariate partial eta squared = 0.55. Body appreciation scores at T1 (*M* = 3.2, *SD* = 0.7), T2 (*M* = 3.6, *SD* = 0.5; *p =* 0.11), and T3 (*M* = 3.5, *SD* = 0.7; *p =* 0.21) were not significantly different. This indicates that the intervention did not have a significant impact on body appreciation scores in the TG.

#### 3.2.3. Intuitive Eating

There was a significant effect for time in an ANOVA with intuitive eating score as the dependent variable, Wilks’ Lambda = 0.21, F (2, 5) = 9.39; *p =* 0.02, multivariate partial eta squared = 0.79. T1 intuitive eating score (*M* = 2.7, *SD* = 0.6) was lower than T2 (*M* = 3.5, *SD* = 0.6; *p =* 0.02) and T3 (*M* = 3.4, *SD* = 0.8; *p =* 0.01). The T2 score was not significantly different than the T3 score (*p =* 1.00). This finding suggests that the intervention promoted a significant increase in intuitive eating scores within the TG and that the increase was maintained at the five-week follow-up.

### 3.3. Question 3: Did the Intervention Produce Similar Changes in Outcome Variables during the Intervention in the WLCG?

#### 3.3.1. Dietary Restraint

A paired-samples *t*-test was conducted to evaluate the impact of the intervention on dietary restraint scores in the WLCG during their intervention period. There was a statistically significant decrease in dietary restraint scores from T2 (*M* = 58.73, *SD* = 21.00) to T3 (*M* = 36.51, *SD* = 16.00), *t*(6) = 4.90, *p* = 0.003 (two-tailed). The mean decrease in dietary restraint scores was 22.22 with a 95% confidence interval ranging from 4.54 to 11.12. The eta squared statistic (0.80) indicated a very large effect size. This suggests that the intervention was able to produce replicable effects on dietary restraint scores in a WLCG.

#### 3.3.2. Body Appreciation

A paired-samples *t*-test was conducted to evaluate the impact of the intervention on body appreciation scores in the WLCG during their intervention period. There was a statistically significant increase in body appreciation scores from T2 (*M* = 3.10, *SD* = 0.57) to T3 (*M* = 3.76, *SD* = 0.57), *t*(6) = −3.308, *p* = 0.016 (two-tailed). The mean increase in body appreciation scores was 0.66 with a 95% confidence interval ranging from −1.14 to −0.17. The eta squared statistic (0.65) indicated a large effect size. This suggests that the intervention was able to produce improvements on body appreciation scores in a WLCG.

#### 3.3.3. Intuitive Eating

A paired-samples *t*-test was conducted to evaluate the impact of the intervention on intuitive eating scores in the WLCG during their intervention period. There was a statistically significant increase in intuitive eating scores from T2 (*M* = 2.88, *SD* = 0.27) to T3 (*M* = 3.82, *SD* = 0.22), *t*(6) = −11.008, *p* < 0.001 (two-tailed). The mean increase in intuitive eating scores was 0.94 with a 95% confidence interval ranging from −1.15 to −0.73. The eta squared statistic (0.95) indicated a very large effect size. This suggests that the intervention was able to produce replicable effects on intuitive eating scores in a WLCG.

## 4. Discussion

This study provides preliminary data suggesting that an intuitive eating intervention may help college students decrease dietary restraint and increase body appreciation and intuitive eating in female-identifying undergraduates. Although there were no differences between groups, which is further discussed in the limitations section, within subjects analyses yielded significant, favorable improvements for dietary restraint and intuitive eating, but inconsistent results for body appreciation, during the intervention period for the TG and during the intervention replication for the WLCG. Finally, data from the five-week follow-up in the TG showed that this impact was sustained.

The within-subjects reductions in dietary restraint seen in the present study are important because research indicates the lack of sustained long-term benefits of dieting and highlights the need for a shift in how healthy eating and weight management are encouraged [35,36,37,38,39]. Utilizing an intuitive eating approach to teach female-identifying undergraduates about healthy eating may help reduce dietary restraint and, in turn, reduce the prevalence of disordered eating practices that develop into eating disorders.

Although improvements in body appreciation were inconsistent in this study, changes were in the expected direction for both groups. These results are important to disordered eating research because other studies have found body appreciation to be inversely related to disordered eating [17]. A study assessing body appreciation and intuitive eating in individuals with a history of an eating disorder proposed that intuitive eating could help foster greater body appreciation because intuitive eating focuses on internal cues and listening to one’s body [40].

This study found improvements in intuitive eating, which were sustained at the follow-up in the TG. These results are important because a review of internal-cues-focused interventions found that intuitive eating may decrease disordered eating behaviors and dietary restraint [41]. More recently, a longitudinal assessment of intuitive eating found that greater intuitive eating and greater increases in intuitive eating scores over an eight-year study were associated with lower odds of engaging in disordered eating behaviors, making intuitive eating an important target for interventional research [42]. Moreover, body appreciation and intuitive eating both appear to have protective influence on disordered eating onset and therefore present as important targets in programs aimed to prevent disordered eating behaviors [43], like the current study. The findings in the present study are consistent with an intuitive eating intervention published by Burnette and Mazzeo, which found reductions in disordered eating and improvements in body appreciation and intuitive eating from pre- to post-test and maintenance at follow-up [23]. Intuitive eating may be beneficial in promoting healthful eating behaviors in college students due to its focus on tuning into one’s internal cues. Dieting removes one’s ability to detect true hunger and fullness as it relies on external cues that dictate what, when, and how much to eat. This can disconnect one from their internal cues and make it harder to feel fullness and stop eating when satisfied. Encouraging intuitive eating principles for college students could be beneficial by teaching individuals to eat when they’re hungry and stop when they’re full.

This study had several strengths and limitations. The most notable strength was that this study utilized a randomized waitlist-controlled design. Thus, it was possible to compare changes in outcomes in a TG during the intervention to a WLCG and evaluate whether the observed effects were replicated in a WLCG. This study was unique by utilizing two Registered Dietitians who received 40 h of training, coaching supervision, and passed an exam to become Certified Intuitive Eating Counselors. Therefore, the intervention facilitators were experts in both nutrition and intuitive eating. The intuitive eating curriculum directly adhered to the principles outlined by Tribole and Resch, which ensured that the intervention sessions matched the underlying theories of intuitive eating that the curriculum was intended to operationalize. This study had low attrition, with only one participant dropping out before receiving treatment due to a scheduling conflict. Lastly, the results of this study demonstrated improvements in both a healthy weight TG and a higher weight WLCG, meaning, improvements were seen despite weight class.

Despite the strengths of this study, there were several limitations. This study utilized self-report measures designed to assess the impact of an intuitive eating intervention. Self-report measures are subject to inaccurate participant responses [44]. Demand characteristics may be at play since the purpose of the intervention was briefly mentioned before the baseline assessment. However, consent was the only time body appreciation and dietary restraint were mentioned as the study’s aims. Future studies should aim to mitigate the effects of potential demand characteristics. The curriculum was tested in a small sample of female-identifying undergraduates who scored high on dietary restraint and low on body appreciation and intuitive eating. Therefore, results may not be generalizable to other populations, such as the general healthy population or those with clinically diagnosed eating disorders. Future research should aim to replicate findings from the current study using a larger sample and comparing an intuitive eating curriculum to an active control, like traditionally used healthy eating and weight management curriculum. Studies should employ a longer follow-up period to study changes in measures over time and longer intervention periods to enhance the learning of the intuitive eating principles. Lastly, future studies should recruit larger sample sizes to extrapolate the effects of intuitive eating interventions on body appreciation.

## 5. Conclusions

This novel study provides important preliminary evidence that an intuitive eating intervention can be used to decrease disordered eating risk factors in female-identifying undergraduates. The study paves the way for future work utilizing intuitive eating as an intervention to improve disordered eating risk factors, thus decreasing rates of disordered eating developing into eating disorders in the female-identifying undergraduate population.

## Figures and Tables

**Table 1:** Weekly breakdown of intuitive eating intervention content including intuitive eating principles taught and session activities.

Week	Principle(s) Taught	Activities
1	Introduction to Intuitive Eating Reject the Diet Mentality	Intuitive Eating Pre-Test Journaling and Meditation
2	Challenge the Food Police Honor Your Hunger Feel Your Fullness	Hunger-Fullness Discovery Scale Journaling and Meditation
3	Make Peace with Food Discover the Satisfaction Factor	Taste Testing Journaling and Meditation
4	Cope with Emotions without Using Food Respect Your Body	Statement Re-Framing Journaling and Meditation
5	Exercise—Feel the Difference Honor Your Health with Gentle Nutrition	Intuitive Eating Post-Test Journaling and Meditation

**Table 2:** Means and standard deviations (SD) of difference scores for cognitive restraint, body appreciation, and intuitive eating across assessment timepoints for the treatment group (TG; *n* = 7) and the waitlist control group (WLCG; *n* = 7).

	T1 to T2 ^a^		T2 to T3 ^b^	
	TG	WLCG	TG	WLCG
Cognitive Restraint Subscale ^c^	−22.2 (19.0)	0.8 (9.3)	4.0 (13.9)	−22.2 (12.0)
Body Appreciation Scale-2 ^d^	0.4 (0.4)	0.1 (0.2)	−0.04 (0.3)	0.7 (0.5)
Intuitive Eating Scale-2 ^e^	0.8 (0.5)	−0.03 (.2)	−0.1 (0.3)	0.9 (0.2)

^a^ T1 to T2 represents baseline to post-intervention for IE1 ^c^ and the waitlist period to baseline for IE2 ^d^. ^b^ T2 to T3 represents the post-intervention to maintenance period for IE1 ^c^ and baseline to post-intervention for IE2 ^d^. ^c^ Cognitive Restraint Subscale items 2, 11, and 12 are rated on a 4-point scale ranging from 4 (“Definitely True”) to 1 (“Definitely False”). Item 15 is rated on a 4-point scale ranging from 1 (“Almost Never”) to 4 (“Almost Always”). Item 16 is rated on a 4-point scale ranging from 1 (“Unlikely”) to 4 (“Very Likely”). Item 18 is rated on an 8-point scale from 1 (no restraint in eating) to 8 (total restraint in eating). Higher scores are indicative of greater cognitive restraint. ^d^ Body Appreciation Scale-2 items are rated on a 5-point scale ranging from 1 (“Never”) to 5 (“Always”). Higher scores indicate a greater positive body image. ^e^ Intuitive Eating Scale-2 items are rated on a 5-point scale ranging from 1 (“Strongly Disagree”) to 5 (“Strongly Agree”). Higher scores on this measure indicate greater levels of intuitive eating.

## Data Availability

The data presented in this study are available on request from the corresponding author. The data are not publicly available due to privacy.
